# SOCAL SAVVY CAREGIVER: FOCUS GROUP INSIGHTS

**DOI:** 10.1093/geroni/igad104.2841

**Published:** 2023-12-21

**Authors:** Iris Aguilar, Maria Aranda

**Affiliations:** Florida International University, Miami, Florida, United States; University of Southern California, Los Angeles, California, United States

## Abstract

Dementia due to Alzheimer’s disease (AD) is a public health crisis in our nation. More than 1.6 million Californians provide unpaid care for a person with AD. Qualitative data from focus group discussions were obtained from unpaid/informal/family caregivers in a clinical trial testing the efficacy-effectiveness of Savvy Caregiver Express compared to the Savvy Caregiver Program.

Objectives: 1) explore class participation motivations, 2) perceived class benefits, and 3) class improvement recommendations.

Methods: We randomly selected and invited participants who had completed the class intervention cycle. Focus group subsample (n=25) was 84% female, mean age 54.48 years (28 to 80 years); 36% non-Latino white, 20% Latino, 8% African American, 4% Asian and American/Pacific Islander. 60% cared for a parent (44% mother and 16% father), 16% cared for a spouse/partner, 20% other relatives, and 4% friends. Focus group were transcribed, coded, and themes were created using inductive analysis.

Findings: Themes identified for discussion topic #1: seeking to gain knowledge about AD and stages, peer support, and caregiver competence; #2 knowing AD stages, management of meaning, caregiving mastery, and self-care; and #3 audiovisuals improvements, class structure changes, printed materials, and ongoing virtual sessions.

Implications: Participation in caregiving classes are critical to the improvement of quality of life for the person with dementia and caregiver. These findings offer actionable changes to improve the transformation of classes and adjust future materials or provide additional resources to help participants fully understand the course and better understand the motivations for increasing engagement with caregivers.

